# Comparing Analysis Methods in Functional Calcium Imaging of the Insect Brain

**DOI:** 10.1371/journal.pone.0129614

**Published:** 2015-06-05

**Authors:** Anna Balkenius, Anders J. Johansson, Christian Balkenius

**Affiliations:** 1 Swedish University of Agricultural Sciences, Alnarp, Sweden; 2 Lund University, Lund, Sweden; 3 Lund University Cognitive Science, Lund, Sweden; University of Arizona, UNITED STATES

## Abstract

We investigate four different methods for background estimation in calcium imaging of the insect brain and evaluate their performance on six data sets consisting of data recorded from two sites in two species of moths. The calcium fluorescence decay curve outside the potential response is estimated using either a low-pass filter or constant, linear or polynomial regression, and is subsequently used to calculate the magnitude, latency and duration of the response. The magnitude and variance of the responses that are obtained by the different methods are compared, and, by computing the receiver operating characteristics of a classifier based on response magnitude, we evaluate the ability of each method to detect the stimulus type and conclude that a polynomial approximation of the background gives the overall best result.

## Introduction

Optical imaging is a technique for measuring neural activity *in vivo* using an activity dependent dye that is applied to the brain and activated using monochromatic light. In insects it is most common to study the olfactory system using this technique. Bath-applied calcium-sensitive dyes have been used in insects to show that odours elicit spatial patterns in the antennal lobe (AL) of the insect brain [1]. The signal is usually very weak depending on the relatively high background fluorescence of cells not contributing to the signal. In the AL, the strongest response is usually about a 1% increase of the signal. In the mushroom body (MB), the signal is an order of magnitude weaker and very sensitive methods are necessary to recover the signal.

Optical imaging presents several obstacles, from the stimulus generation to the processing of the recorded signals. It is necessary that stimuli are precisely timed to the recording of images to allow precise measurements of signal magnitude and latency. Furthermore, it is necessary to cope with noisy signals and to estimate the bleaching of the signal from very few data points to finally recover the response as the difference between the recorded signal and the estimated background normalised by the background (ΔF/F).

To obtain reliable recordings from the AL and MB, we have developed a fully automatic technique that controls every parameter of the stimulus presentation as well as the recording and analysis of the data. The set-up that has been used to record brain activity in insect during multimodal stimulation with visual, olfactory and taste stimuli in several studies [2–6]. The stimulus generation is completely under computer control to allow well timed and repeatable presentations of visual, olfactory and taste stimuli. Here we describe the analysis methods that were developed to estimate the responses to multimodal stimuli. The methods are fully automatic and process every pixel in the image sequence without the need to select regions of interest or set any parameters except for the time window where the analysis should be made, which typically has a fixed relation to the timing of the stimulus presentation.

Many methods for the estimation of the background signal have been reported in the literature. For example, the bleaching curve for control recordings can be used as a background estimate [7]. The average of all image frames [8] or a few frames before the onset of the response [9–11] can be used. In the simplest case, the difference between two frames are used, and the first frame is selected before the response and the second is though to be at the response maximum [12]. Alternatively, the background signal can be obtained low-pass filtering the signal [2]. When the background function is estimated explicitly, the data is used to fit a linear [3, 6] or non-linear [13] function that is assumed to describe the background. It is important that the estimation is done correctly to avoid errors when the background is subtracted. Incorrect estimation has been reported to results in errors as large as 100% [14].

Below, we compare four different methods for the estimation of the background and compare their performance on six data sets to establish the properties and merits of each of the methods. The source code and the data sets are freely available for download.

## Materials and Methods

In this section we describe the different signal processing steps used to analyse the optical imaging data. We present four methods for estimating the background signal and describe the different types of evaluation that will be used to test the different methods.

### Signal Processing

The recorded image sequence consists of a set of image frames *I*(*t*) of size w × h (See Table 1). This image sequence is processed in a number of stages to reveal the response.

**Table 1:** Overview of the data sets used.

	**Data Set**					
	**A**	**B**	**C**	**D**	**E**	**F**
**Species**	*M. sexta*	*M. sexta*	*M. sexta*	*M. sexta*	*C. Pomonella*	*M. sexta*
**Site**	MB	MB	MB	MB	AL	AL
**Animals**	42	23	23	41	30	17
**Recordings**	285	199	193	334	163	51
**Stimuli**	BENZ	PAA	OCT	OCT	PE	PAA
	BENZ+BLUE	PAA+ BLUE	OCT+BLUE	OCT+GREEN	CD	
**Samples**	40/50	40	40	40	40	38
**Image Size**	320×240	320×240	320×240	320×240	320×240	672×512

#### Spatial smoothing

**Fig 1:**
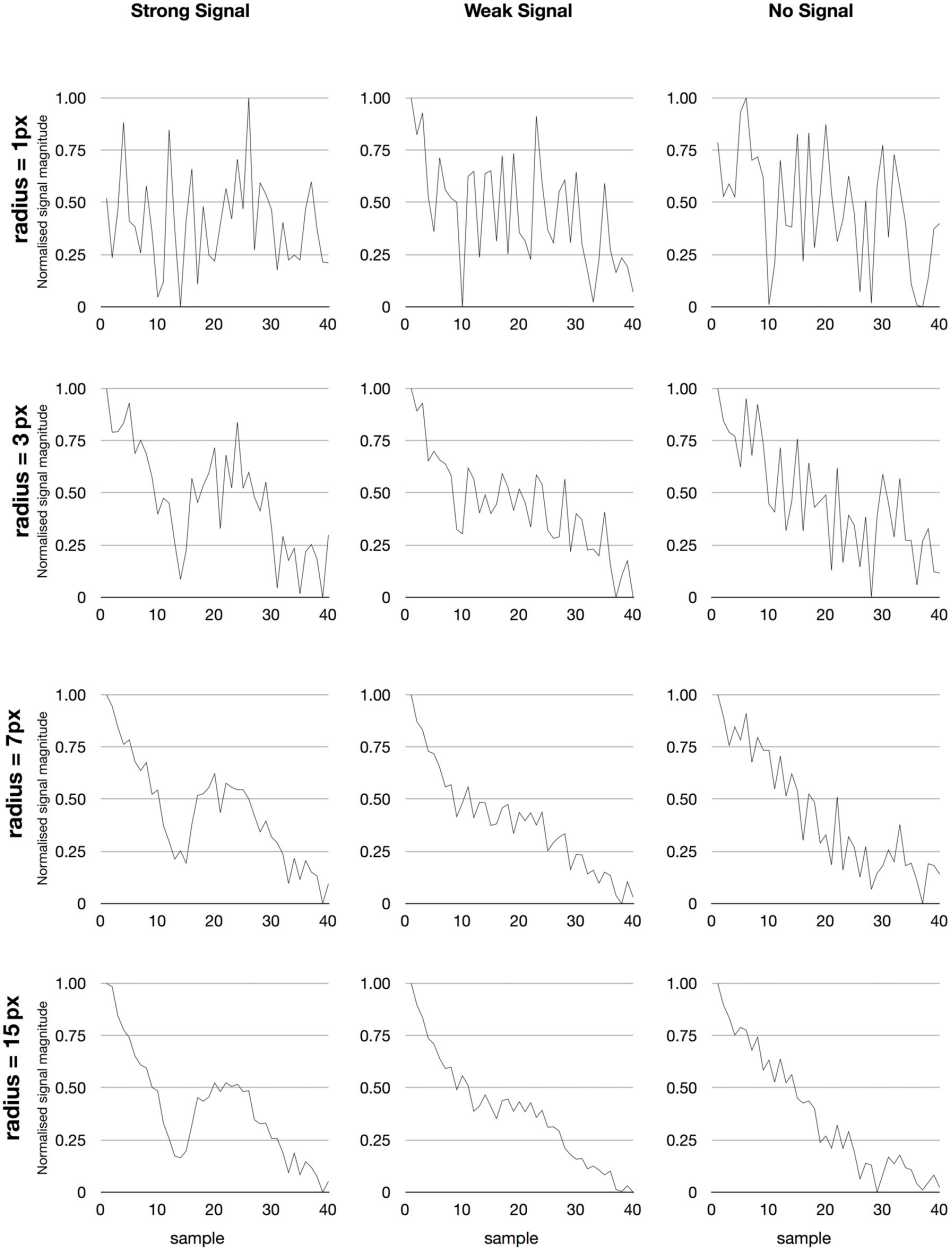
The result of spatial filtering of the recorded signal in a single pixel. The signal in an individual pixel for a single image sequence is very noisy but after spatial filtering the difference between a response and background can clearly be seen. Note that spatial smoothing is used to reveal temporal structure in the signal. The graphs are normalised to more clearly show the result and the filter radius is indicated in pixels. Left. A pixel with a strong response. The response is clearly revealed when a larger area is weighed together. Middle. A weaker signal can only been seen after substantial smoothing. Right. A pixel without a response. Spatial smoothing gradually approximates the bleaching function.

Each frame of the recorded image sequences was processed with a spatial filter to remove noise. This was accomplished by convolving each frame with a Gaussian kernel *G*_*σ*_ with variance *σ*. The value of *σ* is set to obtain the desirable smoothing of the image data (Fig 1) and is kept constant for all recordings within an experiment.

$$\begin{array}{c} S\left(t\right)=I\left(t\right)*G_{\sigma } \end{array}$$ (1)

**Fig 2:**
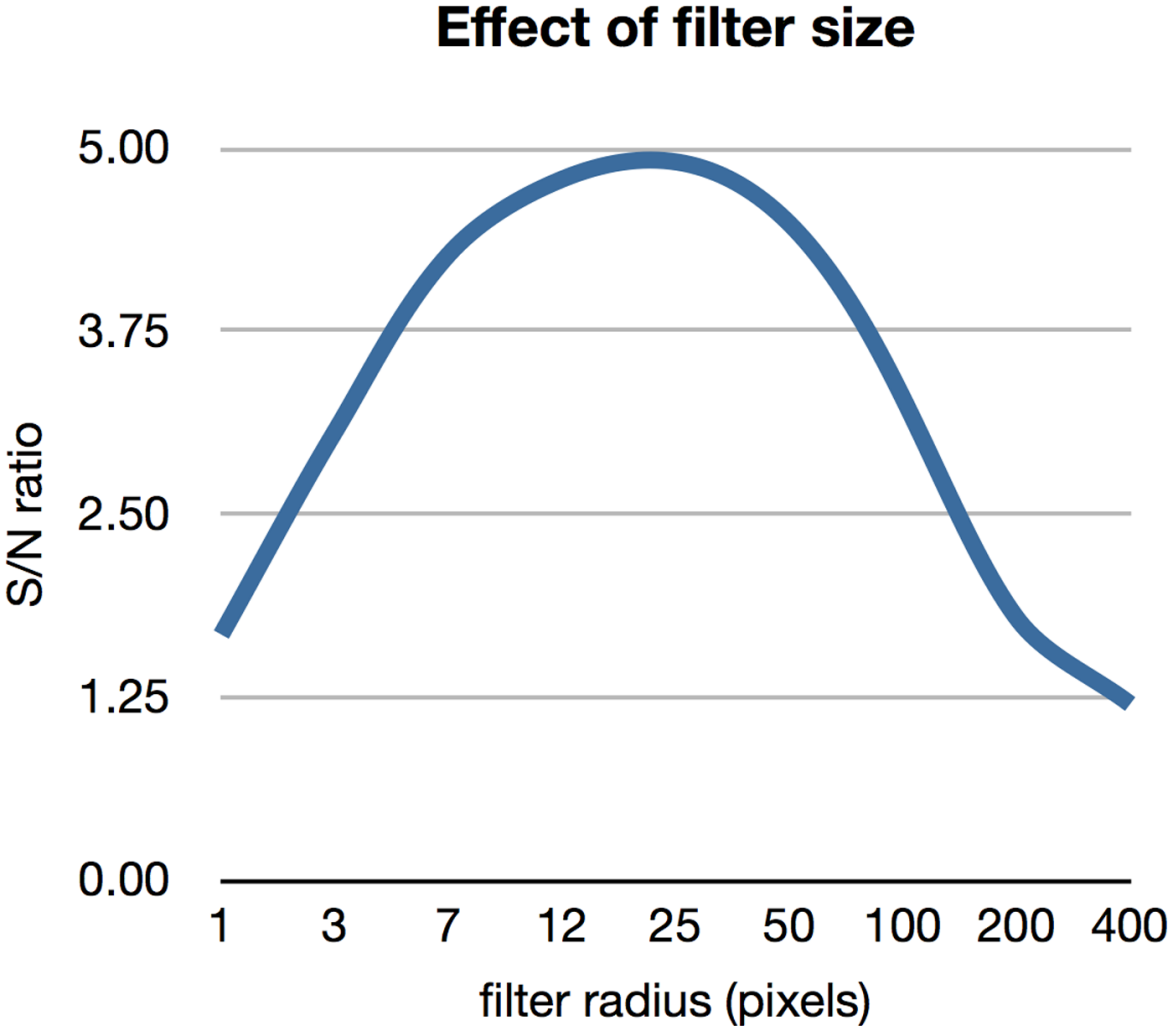
The effect of the filter size on the signal to noise ratio. The signal to noise ratio increases with a larger filter up to a point where it starts to decrease again. Data for a single image sequence.

This operation weighs together the signal at each location with the signal in the surrounding area with a weight that decreases with distance. In addition to removing noise, by changing the width of the Gaussian in this filtering step, it is possible to set whether the analysis will focus on larger or smaller structures. The width of the Gaussian filter is a trade-off between the loss of spatial details in the image and increased signal to noise ratio. Fig 2 shows the signal to noise ratio for a single recording as a function of the filter width. This function can be used to set the width of the Gaussian. Alternatively, the filter width can be guided by the size of the anatomical structures were the signal is assumed to be generated.

Some studies have used a median filter instead of Gaussian smoothing, but such non-linear processing of the image can distort the results and is not necessary with modern cameras where it is very unusual with dead pixels. In contrast, since the integral of the Gaussian filter kernel is 1, Gaussian smoothing only moves activity between pixels and the total signal remains constant.

#### Masking

Before the responses are calculated, the images are first masked to exclude regions that are outside the neural tissue. In general, this has to be done manual, but in our preparation, it is possible to do this automatically by imposing a threshold on the intensity of the images and mask all pixels that are below a certain threshold *T*, a typical choice being T = 33% of the full range. That is, if *S*_*max*_ and *S*_*min*_ are the maximum and minimum pixel values, then all pixels *S*_*x*,*y*_ for which

$$\begin{array}{c} \frac{S_{x,y}-S_{min}}{S_{max}-S_{min}}<T \end{array}$$ (2)

are masked in the final result.

#### Estimating Δ*F*/*F*

After spatial processing, the image signal is processed to estimate Δ*F*/*F*, that is, the difference in signal compared to the background normalised by the background intensity. This computation is complicated by the fact that we do not know the background signal that would have resulted if there were no response. Instead, it is necessary to estimate this background from the available data. In the literature description of background subtraction from calcium evoked responses typically lack detail on the exact methods used in normalising and estimating changes in background intensities.

**Fig 3:**
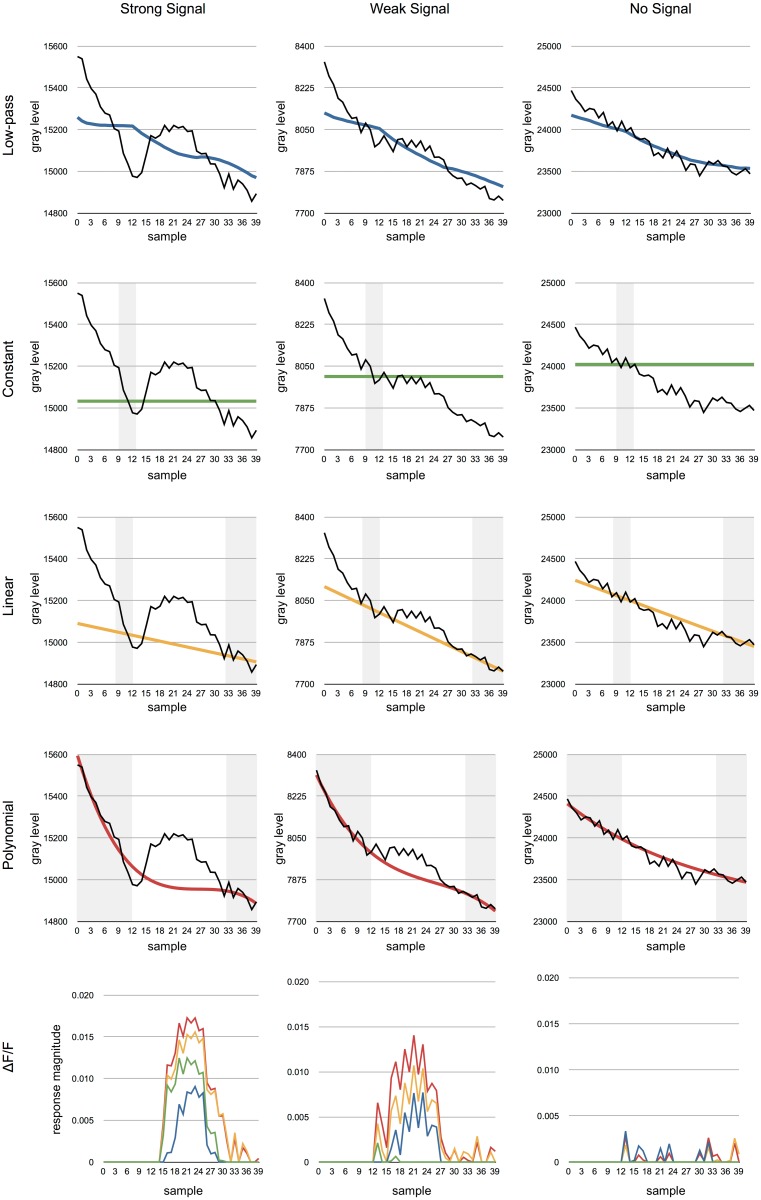
The results of applying the different methods to the example data from Fig 1. The grey regions indicate data used for the approximation of the background function *F* which is shown in different colours. The black line shows the raw signal. Left column. All of the methods are able to recover the signal when it is sufficiently strong. Center column. A weaker signal will be lost if a constant background is used. Right column. None of the methods produce significant responses when the is no signal. Bottom row. The recovered responses based on each of the different methods. The colours are the same as used in the top four rows (blue: low-pass, green: constant, yellow: linear, red: polynomial).

We investigated four methods for estimating the background that result in different results (Fig 3). Depending on the desired accuracy, each of of these methods can be used, but we show that some have clear advantages compared to the others (See Results section).

Although it is possible to use the same background function for each pixel in the image and only scale it with the intensity [13], we prefer to estimate the decay in each pixel individually to compensate for variations over the recorded image.

#### A. Constant

The simplest method is to assume that *F* is constant, that is, that there is no bleaching [8, 10, 11]. Although this is typically not correct, this method can still work well if *F* is assumed to be the level of the signal just before stimulus on-set. If the stimulus starts at frame *i*, the estimation of *F* can be set to

$$\begin{array}{c} \hat{F}=\frac{1}{n}\sum_{k=i-n}^{i}S(k) \end{array}$$ (3)

where *n* is a window before stimulus onset from which the background signal is averaged. This method has also been used to find a first approximation to the extend of the response before being refined by other methods [15].

#### B. Low-pass filter

Another approach is to calculate the background as smoothed versions of the input signal [2], essentially implementing a low-pass filter.

$$\begin{array}{c} \hat{F}\left(t\right)=\frac{1}{n}\sum_{k=t-n}^{t}S(k) \end{array}$$ (4)

#### C. Linear approximation

Alternatively, it is possible to assume that the bleaching is described by a linear function [3, 6]. In this case, a linear function fitted to the recorded signal before and after the respons can be used to estimate the background. Although it is clear that the decay function is not linear, this method works well in practice if the data points used to approximate the decay functions are selected in a suitable way.

The linear background function must be estimated in each pixel since it will be different in different areas of the recorded image. For each individual pixel *I*_*x*,*y*_, the background *F*_*x*,*y*_, is estimated as,

$$\begin{array}{c} {\hat{F}}_{x,y}\left(t\right)=\alpha_{x,y}+\beta_{x,y}t \end{array}$$ (5)

where *α*_*x*,*y*_ and *β*_*x*,*y*_ are calculated using the least square method. Using this method, the initial decay of the signal must be discarded to not distort the result or be taken as a response.

#### D. Non-linear approximation

Given the shape of the bleaching process, the most obvious choice of background function would be to approximate it using an exponential decay function. However, the initial slope of the decay is faster than exponential and if this method is to be used, the initial data must be discarded [13]. A more accurate method would be to use a sum of *n* exponentials to model the background.

$$\begin{array}{c} {\hat{F}}_{x,y}\left(t\right)=\sum_{k=1}^{n}\alpha_{k,x,y}e^{\lambda_{k,x,y}t} \end{array}$$ (6)

Although there exist good methods for estimating the parameters of multi-exponential decay curves [16], they require a larger set of data points than is available here. With a small number of samples, the problem is very ill conditioned and often fails unless the process is manually tuned [17]. This is a well known problem when trying to fit a sum of exponentials to any noisy function with few samples [18], and because of this, we have not used this method. Instead, we have opted to approximate the bleaching using a polynomial. In this case, the background can be estimated by fitting a polynomial to the signal before and after the potential response. We have obtained successful results with a third order polynomial function.

$$\begin{array}{c} {\hat{F}}_{x,y}\left(t\right)=\alpha_{x,y}+\beta_{x,y}t+\gamma_{x,y}t^{2}+\delta_{x,y}t^{3}, \end{array}$$ (7)

where *α*_*x*,*y*_, *β*_*x*,*y*_, *γ*_*x*,*y*_ and *δ*_*x*,*y*_ are estimated constants. As for the linear case (C), the constants can be easily estimated using the least square method and the results will be virtually indistinguishable from that obtained using a hand-tuned sum of exponentials.

#### Calculating the response

Once the background has been estimated, it is used to calculate the signal in each pixel as

$$\begin{array}{c} R_{x,y}\left(t\right)=\frac{S_{x,y}\left(t\right)-{\hat{F}}_{x,y}\left(t\right)}{{\hat{F}}_{x,y}\left(t\right)} \end{array}$$ (8)

Fig 3 shows the results of the different methods on the two signals from Fig 1. Although either method work well in many cases, the recovered signal strength is very different in the four cases. The most accurate response is detected using the polynomial approximation.

#### Signal parameters

**Fig 4:**
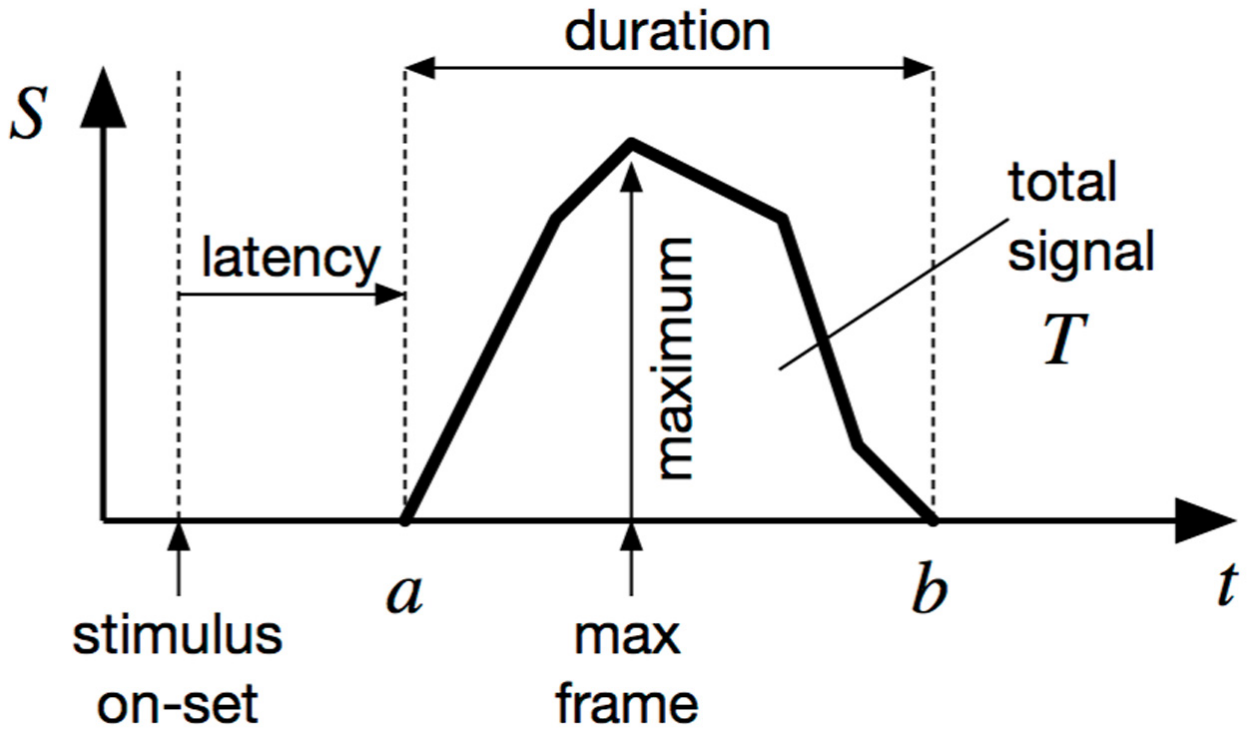
The signal parameters that can be obtained from the processed signal. See text for explanation.

When the signal over time in each pixel has been calculated, a number of parameters of the signal can be calculated (Fig 4). These different measures can be calculated in every pixel or averaged over larger regions.

The magnitude of the response if given by the area indicated by total signal in Fig 4. It is calculated by summing the signal during within a time window where the response is likely to occur. Such a time window is necessary to exclude the beginning and end of the processed signal that will typically include artefacts caused by the background estimation. The average response *A*_*x*,*y*_ over the whole duration of the response has the advantage that it is independent of the sampling rate.

$$\begin{array}{c} A_{x,y}=\frac{1}{\left(b-a\right)}\sum_{t=a}^{b}R_{x,y}\left(t\right) \end{array}$$ (9)

The maximum of the signal can also be determined as well as the corresponding frame.

The signal latency is calculated as the time between the on-set of the stimulus and the start of the response. The respons is assumed to have started on the first frame where it is above zero. This allows the latency to be calculated with the same resolution as the sampling rate. If higher resolution is desired, it can be estimated by assuming the increase in the signal is linear between two frames. If *a* is the index of the first frame with a positive response, then the start of the signal can be calculated as

$$\begin{array}{c} \left(a-1\right)+\frac{R(a-1)}{R(a)-R(a-1)} \end{array}$$ (10)

Similarly, the duration of the signal is given by the time between the start and end of the response. Although the start of the signal is mostly well defined, it can be less clear exactly when the signal ends and it is often useful to introduce a threshold below which the response is assumed to have ended. The exact timing can be calculated in the same way as the start of the signal.

Because there can be variations between the ranges of the signals from different animals depending on the preparation, it can sometimes be useful to normalise the recorded signals for each animal before statistical analysis. This can be done by subtracting the median value and dividing with the average of the two quartiles. Such a normalisation step will put the signals from every animal in roughly the same range while not being overly sensitive to outliers.

### Data Sets

To compare the different models we used six data sets of optical imaging sequences recorded from the antennal lobe and mushroom body in two species of moth (Table 1).

All data sets were recorded using an Olympus microscope (filter settings: dichroic: 500 nm; emission LP 515 nm). The preparation was illuminated at 475 nm and responses were recorded through a 10× (data set A-D, F) or 20× (data set E) objective (NA 0.50; Olympus, Hamburg, Germany). TILL Photonics imaging software (Gräfling, Germany) was used to record the brain responses. The recorded image sequences were stored as 16 bit multi image TIFF files before they were analysed by the image processing software. The details of the preparations can be found elsewhere [2, 6].

#### Data Set A

The first data set consists of recordings from the mushroom body of *Manduca sexta* during presentation of olfactory or multimodal stimuli [2]. This data set consists of 317 recordings where the animal was exposed to benzaldehyde (BENZ) with or without simultaneous visual stimulation with blue light. Each individual image sequence consisted of a minimum of 40 frames (4 Hz, 200 ms exposure time). It has earlier been shown that there were no significant differences in the responses to unimodal and multimodal stimuli for this data set [2].

#### Data Set B

The second data set was recorded in the same species and site as set A. It consists of 133 recordings from 23 animal exposed to phenylacetaldehyde (PAA) with or without simultaneous visual blue stimulation. Each individual image sequence consisted of 40 frames (4 Hz, 200 ms exposure time). There where significant differences in the responses to unimodal and multimodal stimuli for this data set [2].

#### Data Set C

The third data set was also obtained from the mushroom body of *M. sexta* and consists of 87 recordings from 23 animals using 1-octanol (OCT) and a blue colour stimulus [2]. Each individual image sequence consisted of 40 frames (4 Hz, 200 ms exposure time). Earlier analysis had shows significant differences in the responses to unimodal and multimodal stimuli for this data set [2].

#### Data Set D

The fourth data set consists of 334 recordings from the MB of 41 *M. sexta*. The stimuli used were 1-octanol (OCT) and a green colour. Each individual image sequence consisted of 40 frames (4 Hz, 200 ms exposure time). Earlier studies had show no significant differences in the responses to unimodal and multimodal stimuli for this data set [2].

#### Data Set E

Data set E consists of recording from the antennal lobe of the moth *Cydia pomonella*. 30 animals were used and a total of 163 recordings were made. The stimuli consisted of Pear ester (E, Z)-2,4 decadienoate (PE) or the sex pheromone codlemone (E,E)-8,10-dodecadienol (CD). Stimulation started at frame 12 lasted for 1 s. 40 frames were recorded (4 Hz, 200 ms exposure time). Significant differences in the responses to the two odours in the AL [6].

#### Data Set F

The final data set consists of 51 optical recordings from the antennal lobe of 17 *M. sexta*. The stimuli consisted of PAA with two different concentrations. Stimulation started at frame 12 lasted for 1 s. 38 frames were recorded (4 Hz, 200 ms exposure time).

### Evaluation Methods

To compare the performance of the different methods we computed a number of performance measures. To test the performance of each method on estimating the background, we calculated the mean square error of the fit to the data in two ways. In the first case, we used recordings where no stimulus was presented. In the second, we used recordings were stimuli were presented but only used data points outside the temporal window where a response could occur. The average signal magnitude obtained by each method on each data set was calculated. Only the subset of data point produced by the stimulus with the strongest response were used. Measurements without stimulation were consequently excluded. We also calculated the standard deviation of the magnitude measurements, the latency and duration measurements to look at the consistency of the results for each method.

A final analysis looked at the ability of the obtained signals to predict the stimuli that elicited it. For each data set, we constructed a classifier, the role of which was is to classify the type of the stimulus presented. For data sets A-D the classifier had to distinguish between an odour and a multimodal stimulus. For data set E, it had to determine whether PE or CD was used, and for data set F, it was a asked to differentiate between two concentrations of PAA.

The classifier *c*(*x*) was defined as

$$\begin{array}{c} c\left(x\right)=\{\begin{array}{cc} 1 & \text{if}\;x>\tau \\ 0 & \text{otherwise} \end{array} \end{array}$$ (11)

Here, *x* is was based on the total activity as described in Fig 4 above and *τ* is a threshold. Before applying the classifier, the measurements were normalised as described above. Multiple measurements were aggregated. Given the correct classification given by the $\hat{c}(x)$, we calculated the number of false positive and false negative for different thresholds *τ* to construct a receiver operating characteristic (ROC) curve for each method for each data set [19] Finally, the area under the ROC-curve (AUC) was calculated and taken as an index of the performance of each method [20, 21]

In addition to the data sets described above, we created a surrogate data set that closely modelled Data Set A, except that all responses were artificially introduced (cf. [14, 22]). For each recording in Data Set A, we created an image sequence with the same static background image as the original but with an artificially created bleaching curve and response peak. A variable amount of gaussian noise was also added. This data set was used as a ground truth to test the different methods.

The bleaching curve was calculated as a sum of two exponential decay curves and was manually tuned to closely resemble the average bleaching of the original data set. The response was modelled using a gaussian function centered at frame 20 with *σ* = 0.04. The response magnitude was set to a constant multiplied with the range between the average of the first and last frames of the original image sequence.

The signal magnitude of the artificial stimulus was altered and the area under the ROC-curve was calculated for each condition. We also investigated how the level of added noise would influence the background estimation.

### Implementation

The custom made image and signal processing software runs on OS X and makes heavy use of its optimized math libraries vImage [23] and vecLib [24]. The use of the convolution operations of vImage decreased the image processing time by several orders of magnitude compared to running scalar unoptimised code for Gaussian filtering.

In addition to using the optimised math and image processing libraries, the analysis software also makes use of the Grand Central Dispatch functionality of OS X to parallelize the analysis of several images and image sequences [25]. Running on a 12 core Xeon computer, hyper threading allows the software to obtain a processor utilisation at above 2200%, that is, close to the theoretical maximum of 2400% with 12 processors each running two hyper threads each. This results in a processing speed of approximately 500 image sequences (with 40 samples) per minute.

The source code is freely available for download at GitHub.com/balkenius/optima and is distributed under a GPL licence.

## Results

### Background Fit

The average fit of the background estimation to the recording without stimulus presentations for each of the data sets are summarised in Table 2. As could be expected the fit for the constant approximation is worse for all the data sets followed by the low-pass method. The linear approximation work better and the finally, the polynomial fit is best for all the data sets.

**Table 2:** MSE for the different analysis methods.

	**Data Set**					
	**A**	**B**	**C**	**D**	**E**	**F**
**Low-pass**	13.7	31.1	23.9	14.1	4.5	10.3
*std*	13.3	27.4	13.9	13.5	3.5	7.0
**Constant**	89.4	95.0	67.9	91.9	12.5	24.1
*std*	92.3	91.0	40.5	95.2	9.2	16.7
**Linear**	**2.8**	4.9	2.6	2.9	0.8	1.8
*std*	3.2	4.8	1.6	3.3	0.8	1.5
**Non-linear**	3.2	**1.7**	**1.0**	**3.4**	**0.2**	**0.7**
*std*	7.6	2.7	0.6	7.8	0.1	0.7
**n**	196	63	23	203	26	17

The value for the method with the best fit is indicated in bold face.

The results is similar for the fit of the background approximation to the samples outside the response, except that the linear method performs slightly better than the non-linear method of data set A (Table 3).

**Table 3:** MSE for the different analysis methods calculated using data points before and after the response.

	**Data Set**					
	**A**	**B**	**C**	**D**	**E**	**F**
**Low-pass**	19.6	56.5	37.1	26.2	10.1	16.1
*std*	19.7	52.6	30.2	24.8	6.9	18.7
**Constant**	92.8	143.6	89.7	104.4	14.2	30.7
*std*	110.4	140.9	74.3	109.5	8.6	35.2
**Linear**	**2.5**	5.3	3.8	3.6	2.2	2.7
*std*	3.1	4.9	3.7	4.1	1.6	3.5
**Non-linear**	3.1	**2.2**	**0.9**	**3.0**	**0.2**	**0.4**
*std*	8.8	3.7	0.6	8.4	0.1	0.4
**n**	291	187	186	334	163	51

The value for the method with the best fit is indicated in bold face.

### Magnitude

**Fig 5:**
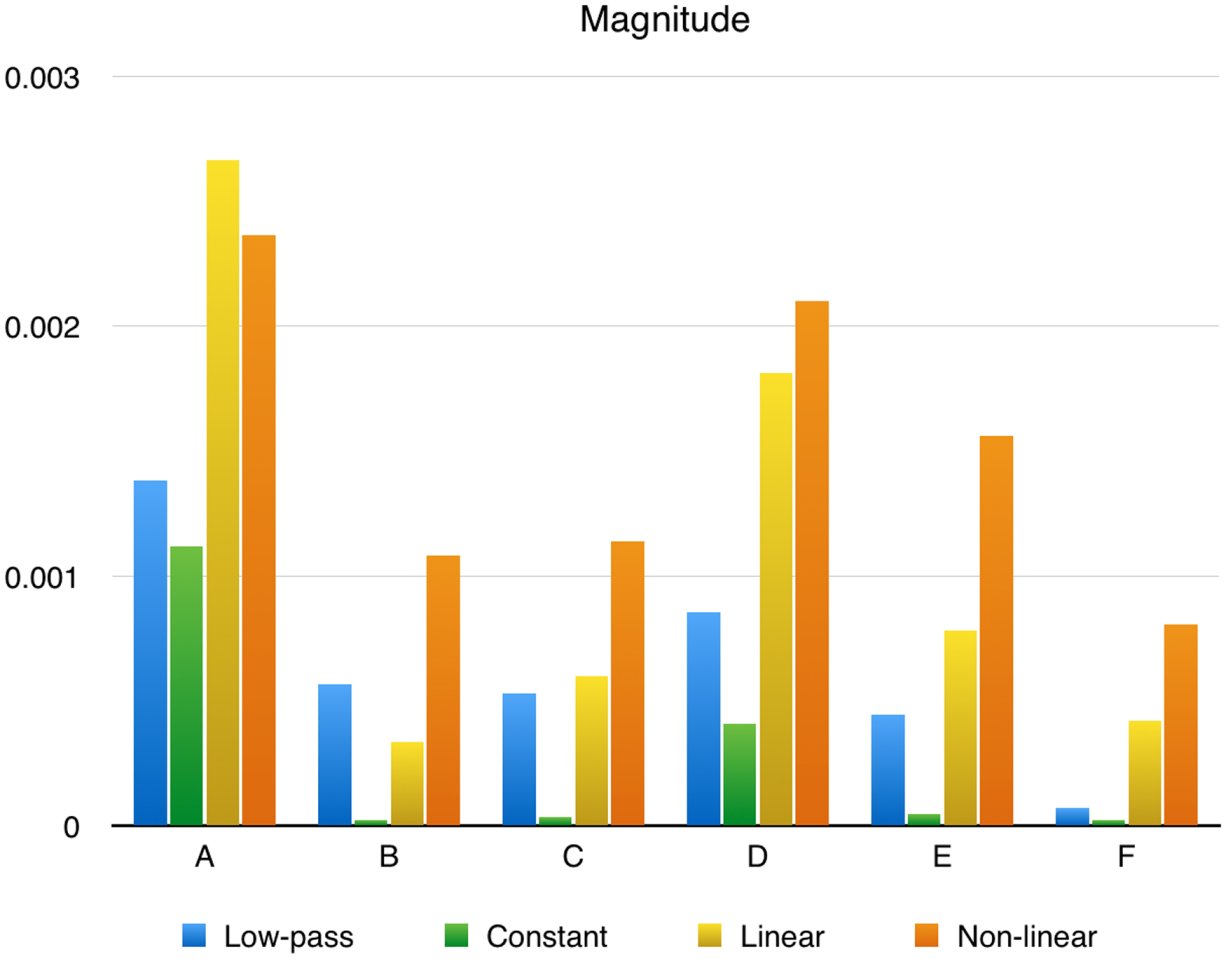
Average response magnitudes. The response magnitude for the different data sets and methods.

The magnitude of the signals obtained using the four methods are shown in Fig 5. The non-linear method consistently produces a larger signal than the other methods closely followed by the linear method. The constant and low-pass method fail to produce any substantial signal on data sets B, C, E, F and F respectively.

### Latency and Duration

The standard deviation of the estimation of latency and duration are presented in Tables 4 and 5. The non-linear method was most consistent in estimating the latency for all data sets except data set F, where the linear method was marginally better.

**Table 4:** Standard deviation of the estimated latency produced by the different methods.

	**Data Set**					
	**A**	**B**	**C**	**D**	**E**	**F**
**Low-pass**	0.90	0.20	0.22	2.00	1.00	2.46
**Constant**	1.33	1.86	1.20	1.82	1.82	1.17
**Linear**	0.96	3.19	3.43	1.98	7.78	2.75
**Non-linear**	0.56	0.35	0.62	1.13	0.82	3.03

**Table 5:** Standard deviation of the estimated response duration produced by the different methods.

	**Data Set**					
	**A**	**B**	**C**	**D**	**E**	**F**
**Low-pass**	2.29	1.82	2.32	2.59	2.83	5.10
**Constant**	5.89	1.45	2.07	4.71	1.55	2.97
**Linear**	0.87	6.26	5.67	4.27	7.37	4.68
**Non-linear**	1.45	1.79	3.16	2.04	1.24	2.74

The results for the estimation of signal duration is less clear (Table 5. Although the non-linear method was best in three cases (data sets B, D, E and F), the linear methods had a lower standard deviation for data set A and the low-pass method was best for data set C.

### ROC Analysis

**Fig 6:**
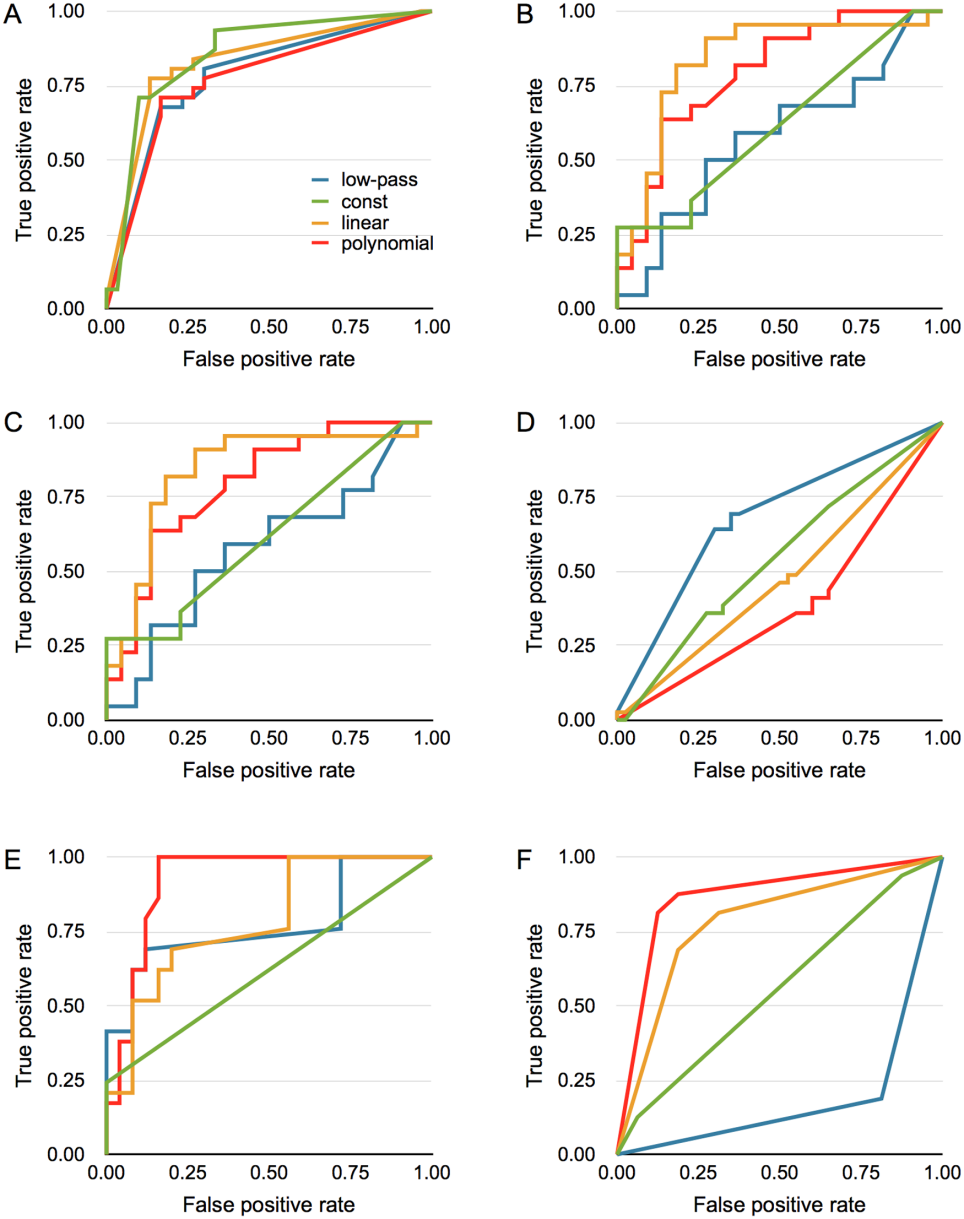
ROC curves for the different methods. The ROC curve for each method and data set indicates who well the differences in response magnitude could be used to distinguish between the two stimulus types described in Table 1.

Fig 6 shows the ROC curves for each data set and method. All methods were able to do well on data set A for Benzaldehyde odour (Fig 6A). For the other data sets, the results were more different for the different methods. For data sets B, C and E, the non-linear method performed best with the linear just behind. For data set D, where there were no significant difference between the response with and without visual stimulation, all methods perform close to chance levels. Finally, for data set F, both the linear and non-linear method perform well while the low-pass method produces a result that is below chance levels. Table 6 shows the area under the ROC curve for each data set and each method.

**Table 6:** Area under the ROC-curve for the different methods and data sets.

	**Data Set**					
	**A**	**B**	**C**	**D**	**E**	**F**
**Low-pass**	0.78	0.58	0.84	0.68	0.77	0.19
**Constant**	0.85	0.62	0.57	0.54	0.62	0.56
**Linear**	0.83	0.84	0.84	0.47	0.78	0.78
**Non-linear**	0.77	0.79	0.82	0.39	0.92	0.87

### Surrogate Data

**Fig 7:**
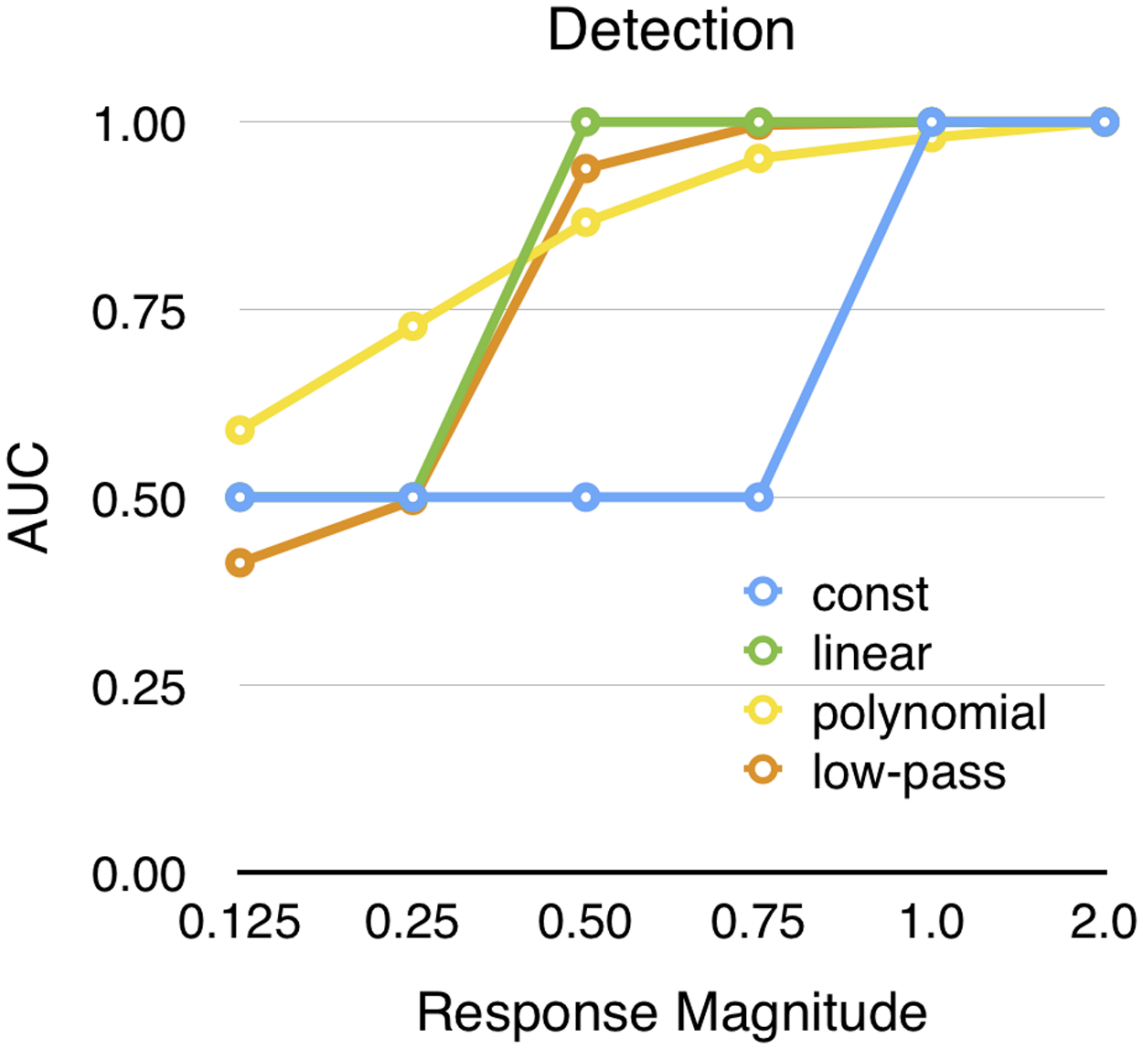
Detection of surrogate data. The area under the ROC-curve was used as an indicator of performance, when the response magnitude was altered.

Fig 7 shows the results on the surrogate data. As could be expected, the signal detecting ability increased with larger response magnitude. The constant, linear and low-pass methods start to classify correctly above a certain signal magnitude. The constant method requires a relatively stronger response. This differs from the polynomial method where the performance increases more gradually. On the other hand, the polynomial method is best at detecting the weaker responses.

**Fig 8:**
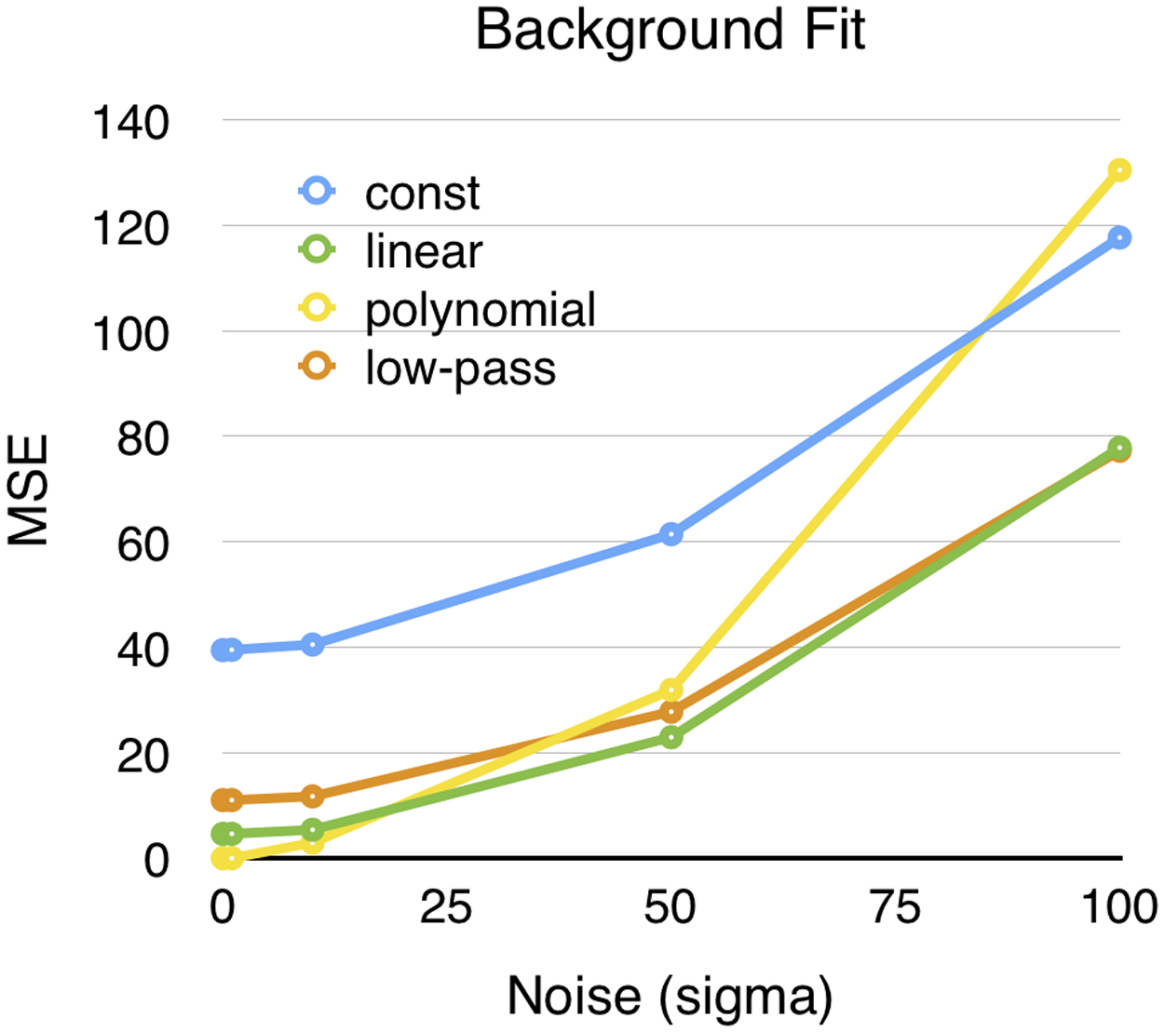
Mean squared error (MSE) of the background estimation on artificial bleaching data with different amounts of noise. A noise level of 25 means that the sigma of the gaussian noise was set to 25 times the range of the bleaching process.

When tested with different amounts of noise, the constant, linear and low-pass methods give different absolute errors, but show similar deterioration with more noise (Fig 8). However, the polynomial method behaves differently. It is more resistant to modest noise levels but has the worst performance with higher levels of noise.

## Discussion

We have tested four signal processing methods on calcium imaging data to obtain the response of the brain in two species of moth to visual and olfactory stimuli. The different methods were tested in both the mushroom body and the antennal lobe.

As expected, the non-linear background estimation resulted in a better fit than a linear, constant or low-pass method (Table 2). This was true for all data sets and is mainly a result of the larger number of freedom for the non-linear approximation.

Looking at the average magnitude of the signal, the non-linear approximation resulted in the strongest signal in all cases with the linear method slightly behind (Fig 5). The commonly used method to average a few frames before the signal on-set and use that as background failed to find any significant response for several of the data sets. This could partly be remedied by setting this value manually for each recoding, but in many cases weak brain response do not produce any positive slope in the signal and a constant background approximation has no chance of retrieving these responses.

The low-pass method has the advantage that it does not rely on a global model of the signal and only filters out local changes. However, it was expected to result in lower signals since it is only sensitive to the faster changes on the signal at the start and end of a response. This is also what was seen in the results.

We also measured the reliability of the different methods to find the latency of the response as well as its duration (Table 4). Here it was predicted that the low-pass method would perform best since it is sensitive to fast changes in the signal. It did indeed work well for all data sets, but the non-linear method was better on four of the data sets. We therefore see no reason to use the low-pass method since it was surpassed by the non-linear method for all other estimates including the estimation of the duration of the responses (Table 5).

The four methods were also tested to see how well they were able to predict the type of stimulus that produced the repsonse. The linear and non-linear background approximations were best in the cases were it could be expected that a classification could be made (Fig 6). For the data set D where there were no significant differences between the multimodal and unimodal responses, all methods performed at close to chance levels (Fig 6D). The only surprising result was data set A where no significant differences were found in the original study but all ROC curves describe a good classification of the different stimuli. The reason for this is the normalisation step used here that was not used in the original study.

The conclusion from the test on surrogate data is that the linear method should be used for data sets with stronger response magnitude while the polynomial method outperforms the other when the response magnitude is lower and there is only limited amount of noise.

While the other methods tend to cut off some of the response, the polynomial method is instead likely to allow some of the noise to be added to the true signal. The reason for this is that the polynomial approximation tend do undershoot the true bleaching curve on the point where its curvature is the largest. Also, when the noise level is very high, the polynomial method is no longer guaranteed to approximate a declining function and this can result in very poor performance as can be seen in Fig 8.

A limitation of the current approach is that each animal is analysed individually. The reason for this is that the Mushroom body of *M. sexta* contains no obvious landmarks that can be used to register recordings from different individuals. If such landmark were available, as in the case for example in the honeybee alfa lobe [26], it is possible to register images from the different animals which would allow for a more straight forward statistical analysis.

Although the non-linear background function is clearly the winner for our data sets, it is in principle possible for data sets to have properties that could be exploited by the other methods, for example if there is more noise later in the signal, that would be discarded by the constant method but would influence the non-linear method. However, we did not see any examples of this in our data. In addition, the ROC-analysis can be used to determine that the analysis method is able to differentiate between the different responses.

The four method we tested have different strengths and weaknesses. The low-pass filtering method has the advantage that it does very few assumptions about the signal. However, it has the disadvantage that the response also contributes to the estimation of the background which will decrease the calculated response. The constant method is simple to use and works reasonably well when the selection of samples to average can be accurately done, but the recovered signal is weaker than that obtained by the other methods in most cases and in the worst case the signal may be completely lost. Using a linear or polynomial approximation gives stronger signals (Fig 5) and better accuracy (Table 6).

We use a polynomial function rather than a sum of exponential for the non-linear background estimation. The problem of fitting a multi exponential to imaging data has been noted before [15], and a simple exponential does not give a very good fit to the data. A multi exponential fit also requires more data than is available (cf. [17, 18]).

Alternative methods for background estimation, like the rolling ball algorithm [27, 28], require that the response only make up a relatively small temporal part of the measured signal and would not be useful for the data used here were the response extends over a larger part of the recorded sequence.

Like other proposed methods [14, 29], our method uses the temporal pattern of the signal to estimate the background, but differs since it addresses a non-stationary background. Furthermore, we do not require regions of interest to be defined. This is useful if the relevant anatomical site is not known or visible in the images. If the site is known, as is the case for most studies of the antennal lobe, the initial spatial smoothing stage can be replaced with averaging over a region of interest covering the site.

An alternative to the background subtraction used here is to directly model both the background and the signal as one parametrised non-linear function [15]. This method is attractive since all the data can be used in the estimation, unlike our methods that can only use data outside the response. However, such methods require a model of the response which may not always be available. Furthermore, the simple exponential decay function used would not fit our data very well as discussed above.

The described analysis steps are sufficiently robust to allow all animals to be included in the analysis. This contrasts with the common practice of removing animals where the signals are either too weak or contain motion artefacts. Our method avoids any such subjective component in the analysis.
